# Erratum to: “Like a lots happened with my whole childhood”: violence, trauma, and addiction in pregnant and postpartum women from Vancouver’s Downtown Eastside

**DOI:** 10.1186/s12954-017-0191-9

**Published:** 2017-09-19

**Authors:** Iris Torchalla, Isabelle Aube Linden, Verena Strehlau, Erika K. Neilson, Michael Krausz

**Affiliations:** 1 Centre for Health Evaluation and Outcome Sciences (CHÉOS), St. Paul’s Hospital, 588-1081 Burrard Street, Vancouver, BC V6Z 1Y6 Canada; 2 Department of Psychiatry, University of British Columbia, Detwiller Pavilion, 2255 Westbrook Mall, Vancouver, BC V6T 2A1 Canada; 3 School of Population and Public Health, University of British Columbia, James Mather Building; 5804 Fairview Avenue, Vancouver, BC V6T 1Z3 Canada

## Erratum

This article [1] was unintentionally published twice in this journal, by the same authors. The following should be considered the version of record and used for citation purposes: “Torchalla et al.: “Like a lots happened with my whole childhood”: violence, trauma, and addiction in pregnant and postpartum women from Vancouver’s Downtown Eastside, Harm Reduction Journal (2014) 11:34, 10.1186/1477-7517-11-34”. The duplicate article [2] is to be ignored. We apologize for not detecting the duplication during the publication process.
